# Decadal evolution of the surface energy budget during the fast warming and global warming hiatus periods in the ERA-interim

**DOI:** 10.1007/s00382-018-4232-1

**Published:** 2019-02

**Authors:** Xiaoming Hu, Sergio A. Sejas, Ming Cai, Patrick C. Taylor, Yi Deng, Song Yang

**Affiliations:** 1 School of Atmospheric Sciences, Sun Yat-sen University, Guangzhou, China; 2 Guangdong Province Key Laboratory for Climate Change and Natural Disaster Studies, Sun Yat-sen University, Guangzhou, China; 3 NASA Langley Research Center, Climate Science Branch, Hampton, VA, USA; 4 Department of Earth, Ocean and Atmospheric Sciences, Florida State University, Tallahassee, FL, USA; 5 School of Earth and Atmospheric Sciences, Georgia Institute of Technology, Atlanta, GA, USA

## Abstract

The global-mean surface temperature has experienced a rapid warming from the 1980s to early-2000s but a muted warming since, referred to as the global warming hiatus in the literature. Decadal changes in deep ocean heat uptake are thought to primarily account for the rapid warming and subsequent slowdown. Here, we examine the role of ocean heat uptake in establishing the fast warming and warming hiatus periods in the ERA-interim through a decomposition of the global-mean surface energy budget. We find the increase of carbon dioxide alone yields a nearly steady increase of the downward longwave radiation at the surface from the 1980s to the present, but neither accounts for the fast warming nor warming hiatus periods. During the global warming hiatus period, the transfer of latent heat energy from the ocean to atmosphere increases and the total downward radiative energy flux to the surface decreases due to a reduction of solar absorption caused primarily by an increase of clouds. The reduction of radiative energy into the ocean and the surface latent heat flux increase cause the ocean heat uptake to decrease and thus contribute to the slowdown of the global-mean surface warming. Our analysis also finds that in addition to a reduction of deep ocean heat uptake, the fast warming period is also driven by enhanced solar absorption due predominantly to a decrease of clouds and by enhanced longwave absorption mainly attributed to the air temperature feedback.

## 1 Introduction

Observational evidence indicates the global-mean surface temperature (GMST) has experienced a relatively rapid warming from the early-1980s to the early-2000s, but has nearly stalled since, producing what is known as the ‘global warming hiatus’ period (Easterling and Wehner 2009; Knight et al. 2009; Liebmann et al. 2010; Solomon et al. 2010; Cowtan and Way 2014; Trenberth 2015). The continuous increase in greenhouse gas concentration is regarded as the key reason for the rapid warming rate in the late twentieth century (Meehl et al. 2007; Randall et al. 2007; Trenberth et al. 2007, 2015; Hansen et al. 2011), but cannot explain the slowdown of global warming since the early-2000s. Since the global warming hiatus period cannot be explained by the greenhouse forcing, the vital impact of internal climate variability on the GMST trend has attracted more and more attention, with a particular focus on the internal climate variability of deep ocean heat uptake (Meehl et al. 2011; Kosaka and Xie 2013, 2016; Trenberth and Fasullo 2013; Chen and Tung 2014; Drijfhout et al. 2014; Marshall et al. 2015) and the effects of internal climate variability on clouds (Hansen et al. 1997; Gregory and Webb 2008; Andrews et al. 2009, 2015; Wild 2012; Wild et al. 2013).

The external forcing and internal climate variability regulate GMST via surface energy flows (Gupta et al. 1999; Smith et al. 2002; Wild et al. 2013). The increase of greenhouse gases drives the fast warming period by enhancing the downward longwave (LW) radiative flux received by the surface (Stephens and Greenwald 1991; Stephens et al. 1994; Iacono et al. 2008; Wang and Dickinson 2013). Another important contributor to the rapid warming is the increase of shortwave (SW) irradiance received by the surface, referred to as the “global brightening” (Wild et al. 2005; Ohmura 2009; Wild 2009, 2012). The increase in shortwave irradiance is closely related to the decrease in cloud cover in the early 1980s (Eastman and Warren 2013). Furthermore, with greater downward SW radiative flux at the surface, the warming effect can be further amplified by the ice-albedo feedback in polar regions, resulting in polar warming amplification especially in the Arctic region (Soden and Held 2006; Bony et al. 2006; Randall et al. 2007; Flato et al. 2013; Taylor et al. 2013). Internal climate variability is also thought to substantially contribute to the rapid warming pace: some studies argue the Atlantic Multidecadal Oscillation (AMO) associated with the thermohaline circulation amplified the surface warming rate as it was in its warming phase during the last two decades of the twentieth century (Wu et al. 2007, 2011; Semenov et al. 2010; Delsole et al. 2011); while others argue the positive phase of the Pacific Decadal Oscillation (PDO) and associated Interdecadal Pacific Oscillation (IPO) accelerated the warming rate during the late twentieth century (Trenberth and Fasullo 2013; Kosaka and Xie 2016; Meehl et al. 2016).

The switch from a positive to a negative phase during the late 1990s of the PDO and IPO is thought to have enhanced the ocean heat transfer from the upper ocean to the deeper ocean (Kosaka and Xie 2013). Ocean heat content showed relatively small decadal changes before 1980, but has increased substantially since (Rhein et al. 2013). The deep ocean heat content (below 700 m), in particular, has increased greatly since the end of the twentieth century, indicating an increase in deep ocean heat uptake. The slowdown of the surface warming during the global warming hiatus period has thus been attributed to interdecadal climate variability through the modulation of the GMST by enhanced deep ocean heat uptake (Meehl et al. 2011; Trenberth and Fasullo 2013; Chen and Tung 2014). Observational global-mean temperature trends also corroborate the important contribution of deep ocean heat uptake to the warming hiatus as the 1–100 m ocean layer has slightly cooled, while the 101–300 and 701–1500 m layers have warmed (Cheng et al. 2015, 2017). Additionally, the global brightening phase of the 1980s and 1990s transitions into a dimming phase in the 2000s in many regions of the world (Norris and Wild 2009; Hayasaka 2016; Wild 2012, 2017), indicating there is a reduction of SW radiative flux received by the surface during the global warming hiatus period. Individual radiative contributions to the surface energy budget from feedback processes including snow and ice cover, water vapor, and ozone may be overwhelmed by contributions from cloud changes (Trenberth and Fasullo 2009). Surface SW flux is further reduced due to volcanic eruptions and increasing aerosol emission (Solomon et al. 2011; Haywood et al. 2014; Santer et al. 2014; Schurer et al. 2015). Changes in aerosol can modify the surface energy budget via direct absorption or scattering of solar energy flux and indirect aerosol–cloud interaction (Ramanathan 2001; Trenberth et al. 2015).

## 2 Simple conceptual picture

**Fig. 1:**
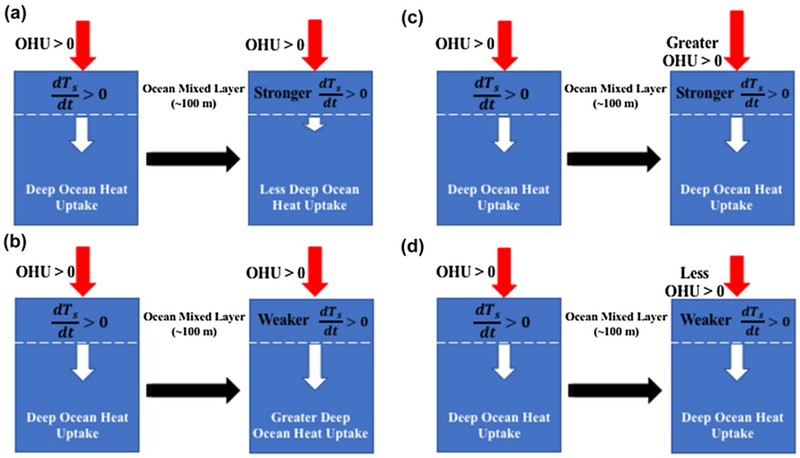
A simple conceptual depiction of how **a** decreases and **b** increases of global deep ocean heat uptake (white arrows) affect the surface warming rate, assuming the ocean heat uptake (red arrows) is constant and positive and there is a background surface warming trend. Similarly, the effects of **c** increasing and **d** decreasing the global ocean heat uptake (red arrows) on the surface warming rate, assuming the deep ocean heat uptake (white arrows) remains constant, are also depicted. Black arrows indicate a change of state

The decadal evolution of global-mean surface temperature is dictated by the net heat intake by the oceans through the surface interface, termed the ocean heat uptake (red arrows in Fig. 1), and the extraction of heat energy from the oceanic surface layer to the deeper ocean layers below, termed the deep ocean heat uptake (white arrows in Fig. 1). Consider a scenario in which the ocean heat uptake is positive (i.e., a net surplus of heat energy into the oceans) and the deep ocean heat uptake is positive (i.e., heat is transferred from the oceanic surface layer to the deeper ocean layers below), the surface temperature can warm, cool, or remain constant depending on the strength of the ocean heat uptake relative to the deep ocean heat uptake; when the ocean heat uptake is greater than (less than) the deep ocean heat uptake the surface warms (cools), but when they are equal the surface temperature remains constant. Let us further consider the climate is experiencing a general long-term positive surface temperature tendency (i.e., a warming trend), corresponding to the scenario that the ocean heat uptake is larger than the deep ocean heat uptake (left blue rectangles of all panels in Fig. 1). As indicated by the right rectangles in each panel of Fig. 1, the strength of the surface warming in the future will then depend on changes in deep ocean heat uptake and ocean heat uptake.

Figure 1 shows four possible scenarios depicting the effects of changes in deep ocean heat uptake and ocean heat uptake on the strength of the surface warming rate. A decrease in deep ocean heat uptake (white arrows in Fig. 1a), assuming a constant ocean heat uptake (red arrows in Fig. 1a), would extract less heat from the oceanic surface layer and thus enhance the surface warming rate (Fig. 1a). Most studies precisely attribute the fast warming period to a reduction of deep ocean heat uptake either in association with the warming phase of the AMO or the positive phase of the IPO (Wu et al. 2007, 2011; Semenov et al. 2010; Delsole et al. 2011; Trenberth and Fasullo 2013; Kosaka and Xie 2016; Meehl et al. 2016). Conversely, an increase of deep ocean heat uptake, assuming a constant ocean heat uptake, extracts more heat from the oceanic surface layer and thus reduces the surface warming rate (Fig. 1b). The decadal increase of deep ocean heat uptake during the global warming hiatus period is thus thought to be the primary contributor to the slowdown of the surface warming in association with a change of phase of the AMO or IPO. On the other hand, if the ocean heat uptake increases with no change in deep ocean heat uptake, the surface warming rate strengthens in response to the greater energy input into the surface layer (Fig. 1c). Conversely, a decrease of the ocean heat uptake, assuming a constant deep ocean heat uptake, would weaken the surface warming rate as less heat energy is provided to the surface layer (Fig. 1d).

As depicted by this simple conceptual picture, in addition to deep ocean heat uptake, decadal changes in ocean heat uptake should also play an important role in establishing the decadal evolution of the global-mean surface temperature. In the remainder of this paper, we explore the role of decadal changes in ocean heat uptake on the fast warming and global warming hiatus periods in the ERA-Interim through a decomposition of the surface energy budget.

## 3 Data and the process-based surface energy decomposition method

All data are monthly mean fields at 1.5° × 1.5° grid boxes covering the globe from 1979 to 2016 and are derived from the latest European Centre for Medium-Range Weather Forecasts (ECMWF) Re-Analysis Interim (ERA-Interim; Dee et al. 2011). The variables include skin temperature, air temperature, specific humidity, cloud cover, cloud liquid water content, cloud ice content, ozone mixing ratio, solar irradiance at the top of the atmosphere (TOA), the downward and the net shortwave and longwave radiative energy fluxes at the surface, surface sensible and latent heat fluxes. The values of the annual mean CO_2_ concentration are set to be the observed 1990 value plus a linear trend as specified in the ERA-Interim (Dee et al. 2011). Note that ERA-Interim used the new climatology for the annual cycle of aerosol distributions derived from Tegen et al. (1997) without inter-annual variation.

Let us consider the time-mean and global-mean surface energy balance equation,

(1) $$OHU=S^{↓}-S^{↑}+R^{↓}-R^{↑}-LH^{↑}-SH^{↑},$$

where *S*^↓^(*S*^↑^) is the surface downward (upward) solar radiative energy flux, *R*^↓^(*R*^↑^) is the surface downward (upward) LW radiative energy flux, *LH*^↑^ and *SH*^↑^ are surface latent and sensible heat fluxes (from surface to atmosphere), respectively, and *OHU* is the ocean heat uptake, measuring the oceanic heat content rate of change in the entire ocean column.

All the terms of (1), except the *OHU* term, can be directly evaluated from the ERA-Interim. Here we use (1) to infer the *OHU* term. The process-based decomposition of the terms on the right hand side of (1) allows us to attribute contributions to the *OHU* by radiative processes and surface turbulent fluxes. To gain confidence in our residual calculation of *OHU* using (1), we compare our residual calculation to the *OHU* directly outputted by the Simple Ocean Data Assimilation (SODA3.4.2), which uses ERA-Interim as the atmospheric forcing. Integration of the *OHU* also allows us to calculate the total oceanic heat content change relative to a base climate state for both the residual calculation of *OHU* and that provided by SODA3.4.2.

We define the decadal mean of 1981–1990 as the base climate state. We construct time series of 24 continuously varying decadal mean anomalies of the global means of all terms in (1), from 1981 to 2014, by taking the difference between the mean of 1982–1991 and the base climate state, the mean of 1983–1992 and the base climate state, and so on up to the mean of 2005–2014 (simply referred to as the “decadal anomalies” hereafter). Using the symbol “Δ” to denote such decadal differences, (1) can be written as follows:

(2) $$\Delta OHU=\Delta S^{↓}-\Delta S^{↑}+\Delta R^{↓}-\Delta R^{↑}-\Delta LH^{↑}-\Delta SH^{↑}.$$

Equation (2) allows us to understand the decadal changes in ocean heat uptake by evaluating the decadal changes in radiative and surface turbulent energy fluxes.

Invoking the linear approximation, we can decompose the sum of the net downward solar radiative energy fluxes at the surface and the downward LW radiative energy flux at the surface into partial radiative energy flux perturbations due to external forcing and individual internal climate processes, namely

(3) $$\Delta S^{↓}-\Delta S^{↑}+\Delta R^{↓}\approx \left[\begin{array}{l} \Delta Q^{Solar}+\Delta Q^{CO2}+\Delta Q^{O3}\Delta Q^{AL}\\ +\Delta Q^{T\_AIR}+\Delta Q^{WV}+\Delta Q^{CL} \end{array}\right],$$

where *ΔQ* denotes the partial radiative energy flux perturbation at the surface due to changes of individual processes where the superscripts “Solar”, “CO2”, “O3”, “AL”, “T_AIR”, “WV”, and “CL” denote the changes, respectively, in the incoming solar energy flux at the TOA, CO_2_ concentration, ozone concentration, surface albedo, air temperatures, water vapor content, and cloud properties. As in Hu et al. (2017), the individual terms on the RHS of (3) are calculated as the difference between two model outputs by the same radiative transfer model (Fu and Liou 1992, 1993): one output uses all inputs from the base climate state, while the other uses identical inputs except for the specific variable (e.g., water vapor) denoted by the superscript, whose input is obtained from one of the 24 decadal mean climate states (i.e., 1982–1991. 1983–1992, …, 2005–2014). The accuracy of the linearization approximation in (3) can be measured by the difference between the right-hand side (RHS) and the left-hand side of (3), which is referred to as the linearization error. Additionally, the radiative energy fluxes derived from the offline radiative transfer model calculations have different values from those directly derived from the ERA-Interim. The difference, namely

(4) $$\Delta Q^{ERR}=\left(\Delta S^{↓}-\Delta S^{↑}+\Delta R^{↓}\right)-{\left(\Delta S^{↓}-\Delta S^{↑}+\Delta R^{↓}\right)}^{ERA}$$

represents the errors in estimating the net surface downward radiative energy flux perturbations, excluding the surface radiative LW emission, by the offline radiative transfer model calculations. Referred to as the offline error, this error is different than the error term due to linearization. The offline error is mainly due to (1) using the mean fields of cloud properties as the inputs of the radiative transfer model to calculate the cloud radiative effects, instead of using instantaneous radiation fields (Taylor et al. 2011; Sejas et al. 2014; Song et al. 2014; Hu et al. 2017), (2) lack of the bias adjustments for aerosol radiative effects in our offline calculation as in ERA-Interim, and (3) applying a different radiative transfer model from that used in the ERA-Interim reanalysis.

The offline calculation error defined in (4) does not include the error in estimating the thermal radiative emission from the surface, which is Δ*R*^↑^ − (Δ*R*^↑^)^*ERA*^. Because the surface thermal radiative emission is primarily dependent on surface temperature (i.e., Stefan–Boltzmann law), since surface emissivity is very close to 1, there is little difference between offline calculations and that derived from the ERA-Interim analysis.

In short, the process-based surface energy decomposition method can be described as

(5) $$\Delta OHU≅\left[\begin{array}{l} \Delta Q^{Solar}+\Delta Q^{CO2}+\Delta Q^{O3}+\Delta Q^{AL}+\Delta Q^{T_{AIR}}+\Delta Q^{WV}\\ +\Delta Q^{CL}-\Delta R^{↑}-\Delta LH^{↑}-\Delta SH^{↑} \end{array}\right].$$

By using (5), the change in the global-mean *OHU* in ERA-Interim reanalysis can be attributed to individual surface energy perturbations due to external forcings (solar and CO_2_) and internal climate processes, including changes in ozone, surface albedo, air temperature, water vapor, clouds, surface temperature, and surface sensible and latent heat fluxes.

## 4 Results

**Fig. 2:**
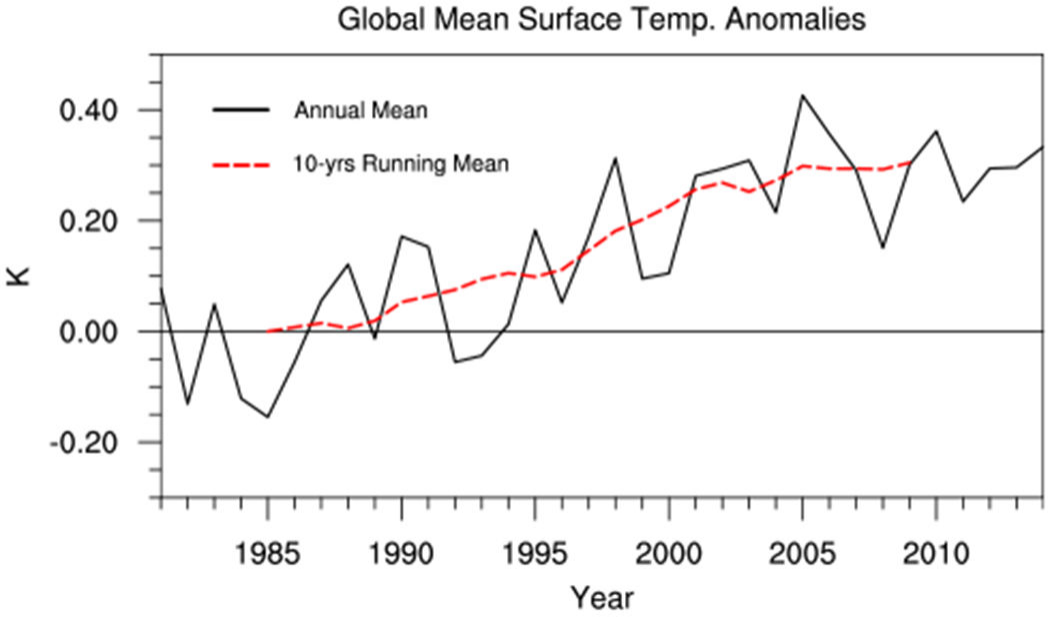
Time series of annual (black solid line) and 10-year running mean (red dashed line) global-mean surface temperature (GMST; K) anomalies from 1981 to 2014 relative to the decadal mean of 1981–1990

The ERA-Interim global-mean surface temperature anomalies relative to the decadal mean of 1981–1990 are shown in Fig. 2. The decadal evolution of the surface temperature anomalies (represented by the dashed red line in Fig. 2) displays a relatively rapid warming during the late twentieth Century, termed here as the ‘fast warming period’, followed by a subsequent slowdown of the surface warming in the early twenty-first century, known as the global warming hiatus period. Below we will compare the decadal changes in *OHU* and the oceanic heat content (which is the temporal integral of *OHU*) with the fast warming and global warming hiatus periods and quantify the processes that are responsible for the decadal changes in *OHU*.

### 4.1 Decadal changes in OHU

**Fig. 3:**
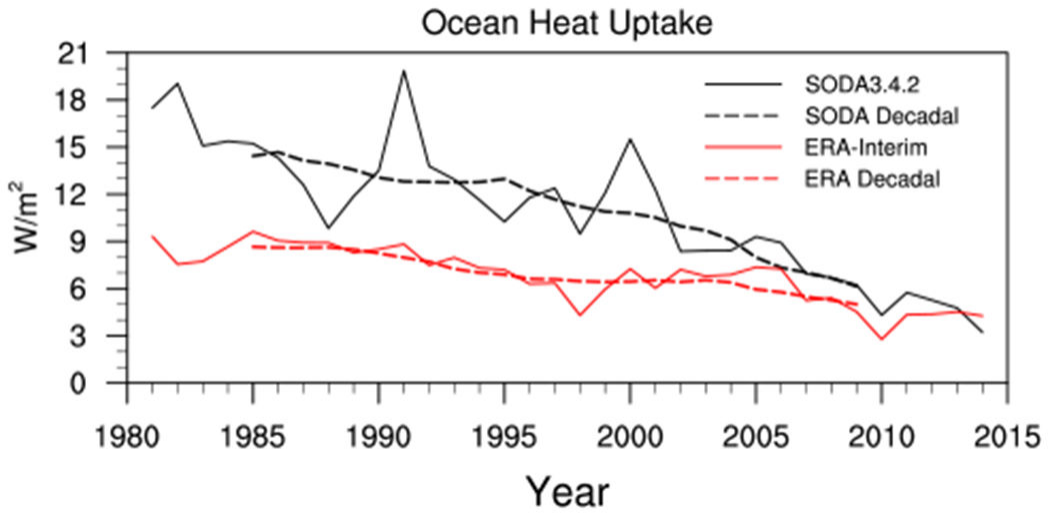
Annual-mean time series (solid lines) and 10-year running mean (dashed lines) of the global-mean ocean heat uptake (W m^−2^) given by SODA3.4.2 (black lines; direct output) and ERA-Interim (red lines; residual calculation)

**Fig. 4:**
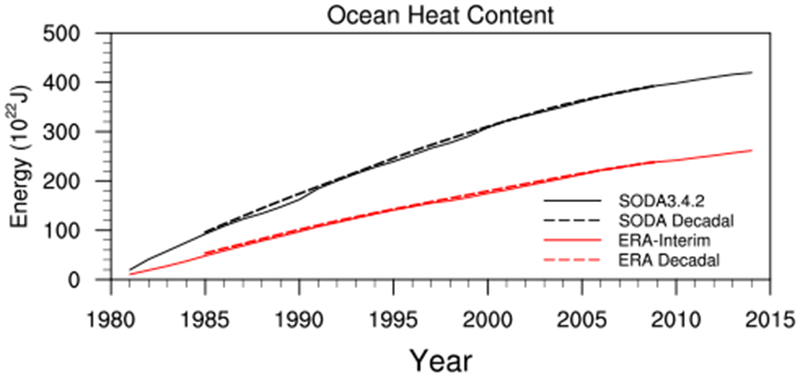
The heat energy (10^22^ J) that has entered the ocean since 1980 as given by SODA3.4.2 (black) and ERA-Interim (red). The 10-year running mean is given by the dashed lines

The dashed lines in Fig. 3 show the decadal evolution of the *OHU* for the whole period of interest (i.e., from 1981 to 2014). We note that the OHU inferred using (1) does seem to underestimate the magnitude of the *OHU* (Fig. 3; red vs black dashed lines), but the sign and general decadal decrease of the *OHU* in the residual calculation is consistent with that given by SODA3.4.2. The positive *OHU* implies there is a net surplus of heat energy into the ocean during both the fast warming and warming hiatus periods. The surplus of heat energy into the ocean increases the total ocean heat content during both periods (Fig. 4), consistent with previous studies (Levitus et al. 2012; Rhein et al. 2013). The decadal decrease of the positive *OHU*, however, indicates the surplus of heat energy into the ocean is decreasing, so the total ocean heat content rate of increase is slowing in the ERA-Interim and SODA3.4.2.

If we assume there is no change in the deep ocean heat uptake, the decadal decrease of *OHU* would imply a slowing of the surface warming. The decadal decrease of *OHU* can therefore help explain the slowdown of the surface warming seen during the global warming hiatus period. The fast warming period, however, cannot be explained by the decrease in *OHU*. Based on the conceptual picture provided in Sect. 2, the strengthening of the surface warming while the *OHU* is decreasing implies the deep ocean heat uptake must be decreasing more rapidly than the *OHU*. The decrease of the *OHU* during the fast warming period thus indirectly corroborates the importance of the decrease in deep ocean heat uptake, as indicated by previous studies, in establishing the fast warming period.

### 4.2 Fast warming period

As indicated by (2), the decadal decrease of global-mean *OHU* is determined by the sum of decadal changes in radiative energy fluxes and surface turbulent energy fluxes. While the (net) *OHU* does not contribute to the rapid warming seen in the late twentieth century, decadal changes in SW radiative flux, LW radiative flux, surface latent heat flux, or surface sensible heat flux, individually, could contribute to the fast warming period.

**Fig. 5:**
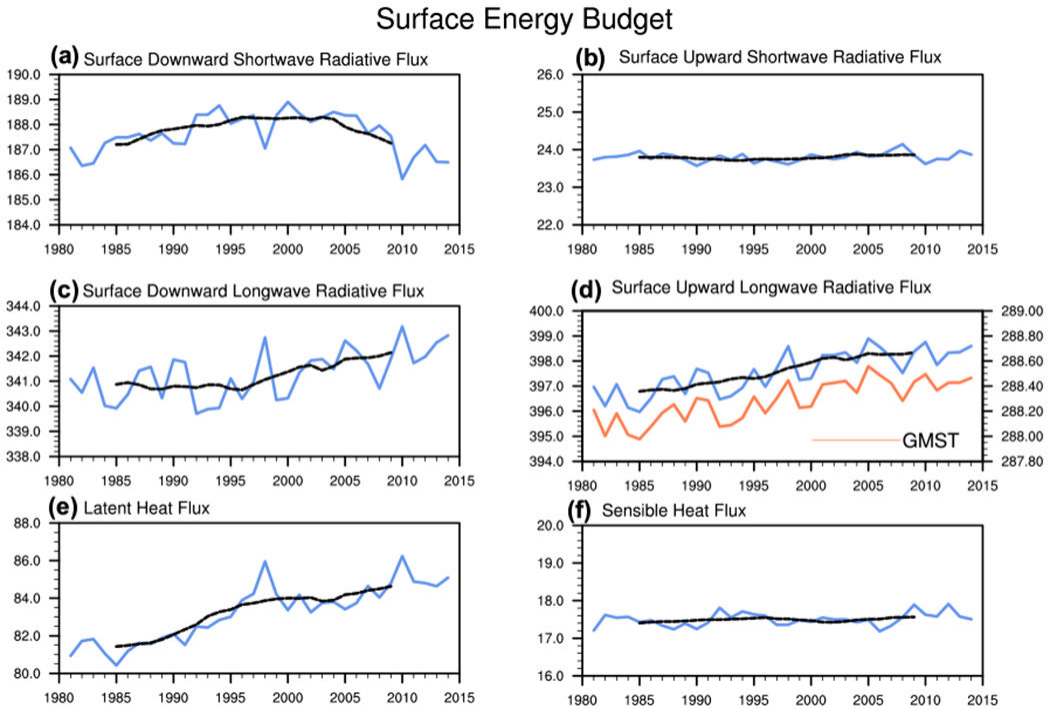
Annual-mean time series (blue curves) and 10-year running mean (black dashed line) of global-mean of **a** surface downward shortwave radiative energy flux, **b** surface upward shortwave radiative flux, **c** surface downward longwave radiative energy flux, **d** surface upward longwave radiative energy flux, **e** surface latent heat flux, and **f** surface sensible heat flux. The orange curve in **d** corresponds to the time series of annual-mean of GMST (K)

**Fig. 6:**
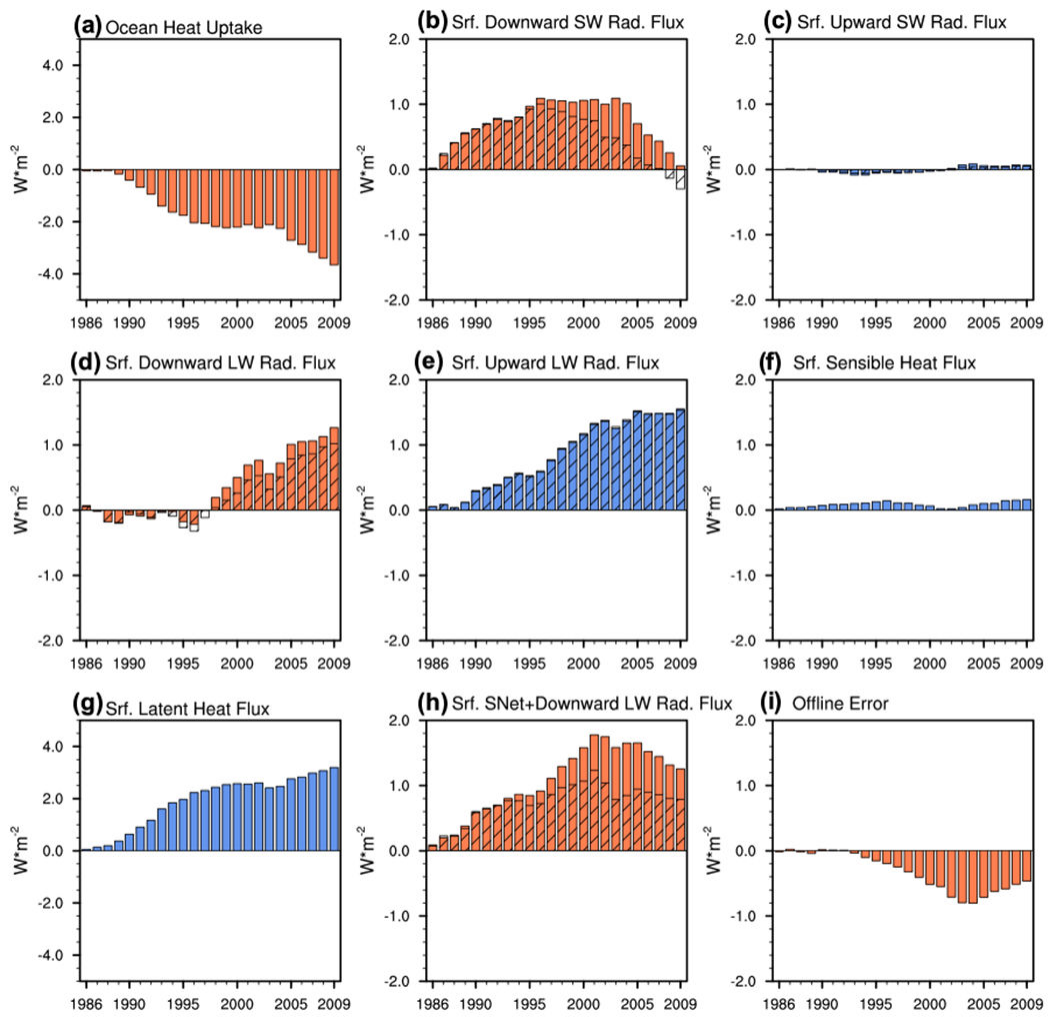
Decadal and global-mean anomalies (W m^−2^) of **a** ocean heat uptake, **b** surface downward shortwave radiative energy flux, **c** surface upward shortwave radiative flux, **d** surface downward longwave radiative energy flux, **e** surface upward longwave radiative energy flux, **f** surface sensible heat flux, **g** surface latent heat flux, **h** the sum of net downward shortwave and downward longwave radiative fluxes at the surface (Δ*Q* = Δ*S*^↓^ − Δ*S*^↑^ + Δ*R*^↓^), and **i** the offline radiative transfer model calculation errors. Positive red (blue) columns indicate an enhanced incoming (outgoing) energy flux. Negative red (blue) columns indicate a reduction of incoming (outgoing) energy flux. The hatched portion of the columns in **b–e, h** is estimated from the offline radiative transfer model calculation and the differences between the full column and hatched portion correspond to the offline radiative transfer model calculation errors

The global-mean downward solar energy flux at the surface (Figs. 5a, 6b) shows a positive trend up to the mid-1990s, which is consistent with the “global brightening” during the period 1980–2000. The global-mean solar energy flux reflected by the surface (Figs. 5b, 6c), however, does not exhibit an easily identifiable decadal trend. As a result, the absorbed solar energy flux at the surface increases during the fast warming period. The increase in solar absorption therefore suppresses the *OHU* decrease during the fast warming period and thus contributes to the fast warming period.

The downward LW flux demonstrates no noticeable trend during early portion of the fast warming period but does increase during the latter part of this period (Figs. 5c, 6d). The downward LW flux increase thus suppresses the *OHU* decrease during the latter part of the fast warming period and contributes to the rapid warming of the surface during this period.

Focusing on the non-radiative terms in the surface energy budget, the global-mean surface latent and sensible heat fluxes show an upward (Figs. 5e, 6g) and nearly flat trend (Figs. 5f, 6f), respectively, during the fast warming period. The surface latent heat flux is the main contributor to the decadal decrease of *OHU*, as the ocean progressively releases greater amounts of latent heat to the atmosphere. The surface latent heat flux increase therefore suppresses the surface warming rate during the fast warming period, while the surface sensible heat flux has a negligible influence.

Following the GMST closely, as expected, the upward LW radiative energy flux rapidly increases during the fast warming period (Figs. 5d, 6e). The increase of the surface LW emission is expected with a positive *OHU*, as the ocean warms in an attempt to balance the incoming surplus of energy. The increase of surface LW emission therefore also contributes to the decadal decrease of *OHU* during the fast warming period.

As previously mentioned, a slowdown of the warming rate during the fast warming period is not observed in parallel to the decrease of *OHU* due to the decrease of deep ocean heat uptake. The greater retention of heat in the oceanic surface layer combined with the increase of surface SW and LW absorption during the fast warming period thus cause the rapid warming of the surface. Furthermore, part of this additional surface energy goes into enhancing evaporation instead of warming the surface. The resultant increases of surface LW emission and surface latent heat flux outpace the increases of surface SW and LW absorption, which explains the decadal decrease of *OHU* during the fast warming period.

### 4.3 Global warming hiatus period

The downward solar energy flux at the surface decreases during the global warming hiatus period (Figs. 5a, 6b), consistent with the “global dimming” scenario reported in Wild (2012). Hayasaka (2016) reports that the decreasing trend of the downward solar flux at the surface after the early-2000s exists in several regions especially in China and Japan, as well as in the tropics (Zhou et al. 2016). Since the decadal upward SW flux change is negligible, the decrease of downward SW radiative flux at the surface also implies a decrease of surface SW absorption. The net downward SW flux decrease thus contributes to the decrease of *OHU* and the slowdown of the surface warming during the warming hiatus period.

The downward LW flux at the surface increases during the global warming hiatus period (Figs. 5b, 6c). The downward LW flux increase thus suppresses the decrease of *OHU* and opposes the slowdown of the surface warming. Combining the net downward SW flux anomalies with the downward LW flux anomalies (Fig. 6h), we see that the decrease of surface SW absorption is stronger than the increase of LW absorption. The net increase of radiative absorption at the surface thus contributes to the decadal decrease of *OHU* and to the slowdown of the surface warming during the global warming hiatus period.

The upward LW flux changes very little during the global warming hiatus period (Figs. 5d, 6e), as expected with the near stalling of the surface warming, and thus contributes minimally to the decadal decrease of *OHU*. Similar to the fast warming period, decadal changes in surface sensible heat flux continue to be negligible during the warming hiatus period (Figs. 5f, 6f), while the surface latent heat flux increases (Figs. 5e, 6g). The surface latent heat flux increase is the primary cause of the decadal decrease of *OHU* witnessed during the global warming hiatus period, and thus contributes to the deceleration of the surface warming.

The reduction of radiative energy absorption at the surface, due to the decrease of downward SW radiative flux, lowers the heat energy surplus into the ocean (i.e., *OHU*). The heat surplus into the ocean that remains goes mainly into enhancing evaporation instead of warming the surface. Combined with the increase of deep ocean heat uptake, as indicated by previous studies, these factors lead to a substantial slowdown of the surface warming, thus causing the global warming hiatus period.

## 5 Process-based radiative decomposition of the surface energy budget

The above results demonstrate the importance of radiation in both contributing to the fast warming and global warming hiatus periods. However, we still do not have a clear grasp of what radiative processes produce the aforementioned radiative effects. We therefore decompose the radiative terms in the surface energy budget to deepen our understanding of the individual processes responsible for the radiative contributions to the fast warming and global warming hiatus periods.

**Fig. 7:**
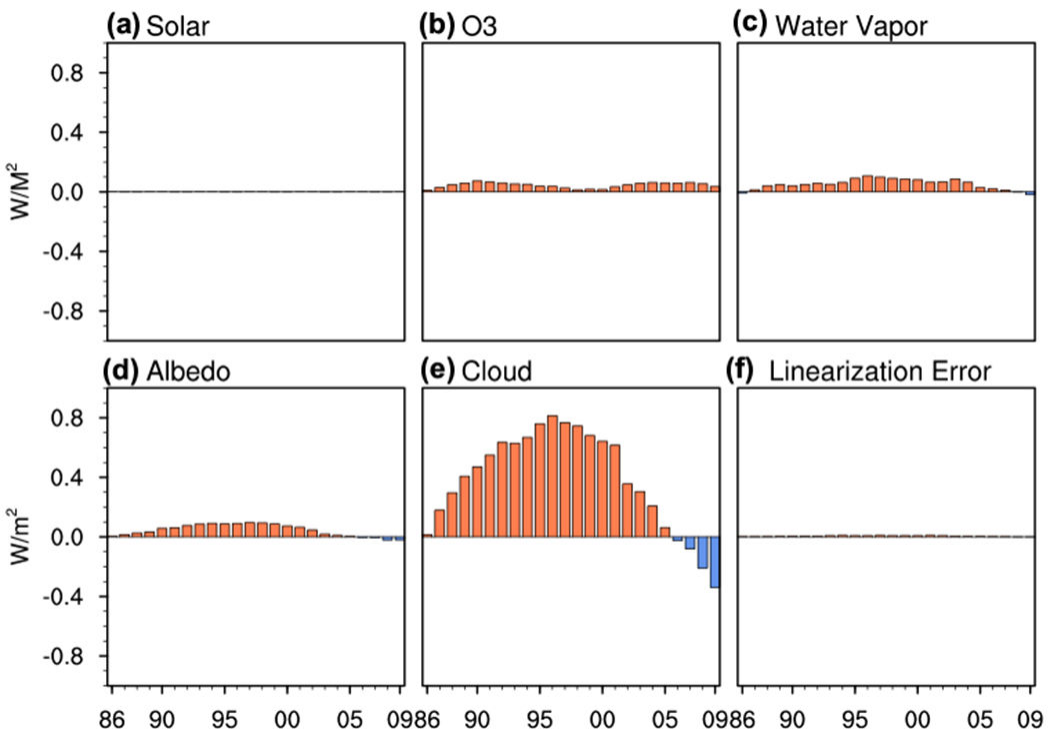
Decadal and global-mean anomalies of net downward SW radiative energy flux (W m^−2^) at the surface due exclusively to changes in **a** solar irradiance at the TOA, **b** ozone, **c** atmospheric water vapor, **d** surface albedo, and **e** clouds. The difference between the sum of panels **a–e** and the sum of the hatched portion of the columns in Fig. 6a, b corresponds to the **f** SW linearization errors in calculating **a–e**

**Fig. 8:**
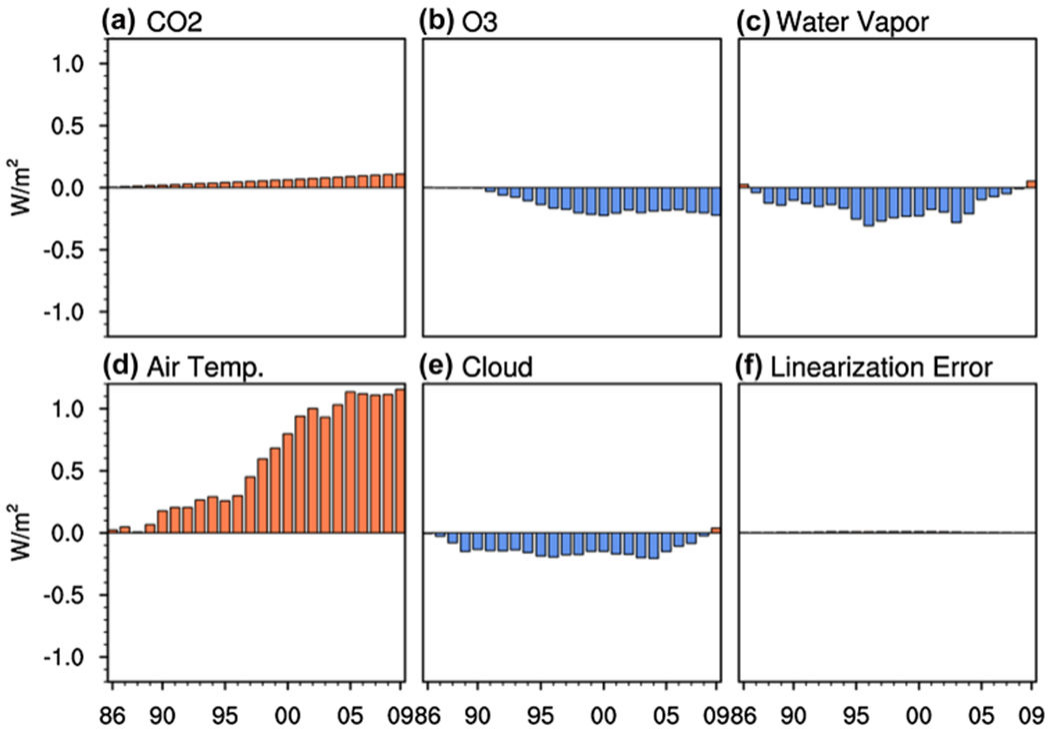
Decadal and global-mean anomalies of downward LW radiative energy flux (W m^−2^) at the surface due exclusively to changes in **a** CO_2_, **b** ozone, **c** atmospheric water vapor, **d** air temperatures, and **e** clouds. The difference between the sum of panels **a–e** and the hatched portion of the columns in Fig. 6d corresponds to the **f** LW linearization errors in calculating **a–e**

We apply (3) to linearly decompose the (total) net downward SW radiative flux and downward LW radiative flux anomalies into net downward SW radiative flux and downward LW radiative flux anomalies due to individual processes. The linearization error can be measured by the difference between the sum of all the individual anomalies and the anomalies derived from the offline radiative calculations. Figures 7f and 8f demonstrate the linearization error is very small, implying that the linear decomposition of the (total) net downward SW radiative flux and downward LW radiative flux anomalies into individual net downward SW radiative flux and downward LW radiative flux anomalies is viable.

Displayed in Fig. 7 are the decadal anomalies of net downward SW radiative energy flux due to individual processes. As there is negligible decadal changes in the incoming solar energy flux at the TOA in the past 37 years, it has no contribution to the net downward SW flux anomalies at the surface (Fig. 7a). Decadal changes in ozone, surface albedo, and water vapor have very small positive contributions to the net downward SW radiative energy flux anomalies at the surface (Fig. 7b–d) and thus contribute very little to the decadal surface temperature variation throughout the whole period between 1981 and 2014. Decadal changes in cloud properties contribute the most to the magnitude of net downward SW flux anomalies at the surface (Fig. 7e). Temporally cloud changes lead to an enhancement of SW absorption up to the mid-1990s followed by a decrease in SW absorption, matching the temporal evolution of the (total) net downward SW radiative energy flux anomalies. The positive trend in net downward SW flux anomalies up to the mid-1990s indicate a decrease in clouds, consistent with the “global brightening” starting in 1980 reported by Wild et al. (2005). After the mid-1990s global cloud water content in the ERA-Interim exhibits a positive trend (Heng et al. 2014); global cloud fraction also increases and has a strong negative correlation with the solar radiative energy flux incident at the surface (Zhang et al. 2016), consistent with the negative trend in net downward SW flux anomalies during this time period. The net downward SW flux anomalies and their decadal evolution are therefore predominantly due to changes in cloud properties.

The SW cloud feedback is thus an important contributor to the rapid warming during the fast warming period and the suppression of the warming during the global warming hiatus period in the ERA-Interim. While cloud changes are known to be a key factor modifying Earth’s energy budget, they are also thought to be the largest source of radiative energy flux uncertainty (Andrews et al. 2012; Zhang et al. 2016; Wild 2017). The temporal variation of clouds in the ERA-Interim, though, is consistent with observational results, such as that given by the ISCCP (Norris et al. 2016), providing a greater degree of confidence in the results.

A decomposition of decadal downward LW flux anomalies is shown in Fig. 8. The direct effect of increasing the CO_2_ concentration during the period from 1980 to 2014 steadily increases the LW absorption at the surface (Fig. 8a) but is not the main contributor to the total downward LW flux anomalies. Overall, the main contributor to the (total) downward LW flux anomalies is the air temperature feedback (Fig. 8d). Decadal changes of ozone, water vapor, and clouds have small individual contributions to the (total) downward LW flux anomalies (Fig. 8b, c, e) relative to the air temperature feedback.

The downward LW flux anomalies due to changes in air temperature have a very similar temporal pattern to the GMST anomalies. As illustrated in Sejas and Cai (2016), lower tropospheric air temperatures and surface temperatures are strongly coupled via the temperature feedback loop (i.e., the thermal–radiative coupling), which explains the matching temporal patterns. Thus, focusing only on the air temperature feedback, one would expect the downward LW flux anomalies to contribute to the fast warming and global warming hiatus periods. However, the combined effects of ozone, water vapor, and clouds negate the effects of air temperature changes until the mid-1990s, which explains the negligible trend in the (total) downward LW flux anomalies up to the mid-1990s. From the late-1990s to the early-2000s, the enhancement of LW absorption at the surface due to warming air temperatures dominates, explaining the positive trend in the (total) downward LW flux anomalies during this period. After the early-2000s the trend in downward LW flux anomalies due to air temperature changes flattens out, so air temperature changes cannot explain the positive trend beyond the early-2000s in the (total) downward LW flux anomalies. Instead, it is the combined increase of downward LW flux from increases in CO_2_, water vapor, and clouds that cause the positive trend in the (total) downward LW flux anomalies after the early-2000s.

## 6 Summary and conclusions

We examined the decadal evolution of the GMST since the 1980s in the ERA-Interim. Consistent with observations (Smith and Reynolds 2005; Hansen et al. 2010; Foster and Rahmstorf 2011; Schmidt et al. 2014) and other re-analysis products (Simmons et al. 2004; Smith et al. 2008; Kosaka and Xie 2016; Hu et al. 2017), a fast warming period is found from the early-1980s to early-2000s followed by a substantial slowdown in the warming, known as the global warming hiatus period.

Decadal changes in deep ocean heat uptake associated with interdecadal oscillations in the ocean (e.g., IPO) constitute the leading hypothesis to explain the fast warming and warming hiatus periods (Wu et al. 2007, 2011; Semenov et al. 2010; Delsole et al. 2011; Kosaka and Xie 2013, 2016; Trenberth and Fasullo 2013; Chen and Tung 2014; Meehl et al. 2016). However, decadal changes in *OHU* also influence the global surface warming rate. A surface energy budget analysis allows us to associate changes in *OHU* with processes that regulate the surface energy balance. Furthermore, a linear decomposition of the radiative terms allows us to attribute individual process contributions (e.g., clouds) to the decadal surface warming rate.

The net downward SW flux increases until the mid-1990s, the increase of SW absorption at the surface thus contributes to the fast warming period. The decomposition of the net downward SW flux anomalies reveals a decrease in clouds is mainly responsible for the enhanced SW absorption during the fast warming period. The increase in downward LW flux from the late-1990s to early-2000s contributes to the latter part of the fast warming period. The decomposition of the downward LW flux anomalies indicates the air temperature feedback is predominantly responsible for the enhanced LW absorption at the surface during the latter part of the fast warming period. Though the downward LW flux and net downward SW flux contribute to an increase of the *OHU*, we see the *OHU* decreases during the fast warming period (Fig. 1). The decadal increase of radiative absorption combined with the decrease of deep ocean heat uptake, which restricts most of the gained energy to the upper layers of the ocean, enhance surface evaporation and surface warming, the latter of which establishes the fast warming period. The decrease in positive *OHU* is thus due to the decadal increases of surface latent heat fluxes and surface upward LW flux, which outpace the decadal increase in incoming radiative energy (i.e., the surface energy budget is closer to reaching a balance).

Since the early-2000s, a slowdown of the surface warming rate has occurred in parallel to the decadal decrease of *OHU*, known as the global warming hiatus period. The decadal increase of the downward LW flux has continued but mainly due to the combined effects of increasing CO_2_, water vapor, and clouds, instead of atmospheric warming. The downward LW flux increase, however, has been offset by the decadal decrease of the net downward SW flux during this same time period due predominantly to cloud increases. The SW effect dominates the LW effect such that the total downward radiative flux has a negative trend and thus contributes to the slowdown of the surface warming. The reduced radiative energy absorbed by the surface goes into enhancing evaporation instead of surface warming, the decadal increase of surface latent heat flux therefore also contributes to the slowdown of the surface warming.

The results of this study are complementary to previous studies that emphasize the role of deep ocean heat uptake in establishing the fast warming and global warming hiatus periods. Even though our results clearly show that the CO_2_ increase is not directly responsible for the fast warming or global warming hiatus periods in the ERA-Interim, this study cannot distinguish whether the contribution of changes in clouds and other climate variables to the fast warming and global warming hiatus periods are triggered by the increase of CO_2_, interdecadal climate variability, or both. The fast warming and warming hiatus periods imply a superposition of the anthropogenic warming signal and climate variability signal, as climate variability on its own would be expected to produce both warming and cooling trends in global-mean surface temperature. Previous studies indicate climate variability has amplified the surface warming during the fast warming period, and suppressed the warming during the hiatus period (Wu et al. 2007, 2011; Chen and Tung 2014; Meehl et al. 2016). The next step would thus be to unravel the connection the changes in climate variables important for the fast warming and global warming hiatus periods, delineated in this study, have with both the increase of CO_2_ and climate variability.
